# Construction of an artificial consortium of *Escherichia coli* and cyanobacteria for clean indirect production of volatile platform hydrocarbons from CO_2_

**DOI:** 10.3389/fmicb.2022.965968

**Published:** 2022-10-21

**Authors:** Yixuan Cui, Faiz Rasul, Ying Jiang, Yuqing Zhong, Shanfa Zhang, Tomasz Boruta, Sadaf Riaz, Maurycy Daroch

**Affiliations:** ^1^School of Environment and Energy, Peking University Shenzhen Graduate School, Shenzhen, China; ^2^Department of Bioprocess Engineering, Faculty of Process and Environmental Engineering, Lodz University of Technology, Lodz, Poland

**Keywords:** ethylene, isoprene, cyanobacteria, co-culture, sucrose, microbial community

## Abstract

Ethylene and isoprene are essential platform chemicals necessary to produce polymers and materials. However, their current production methods based on fossil fuels are not very efficient and result in significant environmental pollution. For a successful transition more sustainable economic model, producing these key polymeric building blocks from renewable and sustainable resources such as biomass or CO_2_ is essential. Here, inspired by the symbiotic relationship of natural microbial communities, artificial consortia composed of *E. coli* strains producing volatile platform chemicals: ethylene and isoprene and two strains of cyanobacteria phototrophically synthesizing and exporting sucrose to feed these heterotrophs were developed. Disaccharide produced by transgenic cyanobacteria was used as a carbon and electron shuttle between the two community components. The *E. coli cscB* gene responsible for sucrose transport was inserted into two cyanobacterial strains, *Thermosynechococcus elongatus* PKUAC-SCTE542 and *Synechococcus elongatus* PCC7942, resulting in a maximal sucrose yield of 0.14 and 0.07       g/L, respectively. These organisms were co-cultured with *E. coli* BL21 expressing ethylene-forming enzyme or isoprene synthase and successfully synthesized volatile hydrocarbons. Productivity parameters of these co-cultures were higher than respective transgenic cultures of *E. coli* grown individually at similar sucrose concentrations, highlighting the positive impact of the artificial consortia on the production of these platform chemicals.

## Introduction

Platform chemicals, defined as essential chemical building blocks serving as precursors to numerous secondary chemicals and materials are essential components of our economy. Ethylene and isoprene are platform chemicals with conjugated double bonds and the primary substrates for the production of many polymers such as synthetic rubber and resins (Easterbrook and Allen, 1987; Deepak et al., 2019). With the advance of industrialization, the demand for basic industrial materials is rising. At present, these two substances are mainly produced from petroleum raw materials, such as natural gas, naphtha, diesel, and heavy oil, which are obtained by petroleum fractionation and petroleum cracking (Valenzuela et al., 2008; Zhang et al., 2020). These methods result in significant carbon dioxide emissions and environmental polluting by-products. Therefore, it is a hot research field to seek a cleaner production method. With biosynthesis technology, the production of these by-products is avoided, and the harm to the environment is reduced. Studies have shown that the two volatile platform hydrocarbons, ethylene, and isoprene, can be produced through biological systems using their corresponding synthases and associated, native biosynthetic pathways. Isoprene in *E. coli* can be synthesized from dimethylallyl pyrophosphate (DMAPP) precursor generated by a native methylerythritol 4-phosphate (MEP) pathway using heterologously expressed isoprene synthase (IspS; Kuzuyama, 2002). Ethylene can, in principle, be synthesized in *E. coli* from methionine by NADH: Fe(II)EDTA oxidoreductase or from α-ketoglutaric acid (AKG) by the ethylene-forming enzyme (Efe; Kazuhiro et al., 1991). The first pathway requires ammonia limitation (C/N = 20) conditions, which is not suitable for the growth of *E. coli* or cyanobacteria; therefore, the pathway utilizing Efe is more optimal (Ishihara et al., 1995). Both precursors, DMAPP and AKG, are readily available in *E. coli,* making it a suitable cell factory for producing these metabolites.

Inspired by the symbiotic relationship of microbial communities in nature (Beliaev et al., 2014), researchers designed and simulated the symbiosis system with artificial substance exchange pathways (Brenner et al., 2008) and gradually developed synthetic consortia between the same microbial species (such as co-culture of multiple *E. coli* strains; Zhang et al., 2022), heterotrophic microbial consortia (such as co-culture of *E. coli* and fungi; Minty et al., 2012), or autotrophic-heterotrophic consortia (such as co-culture of cyanobacteria and heterotrophic *Streptomyces* or *E. coli*; Li et al., 2017; Weiss et al., 2017) and used them in pollution abatement and product biosynthesis (Hays et al., 2016). Studies show that this method avoids the frequent issues caused by some microbial contamination and other undesirable factors associated with cultivating a single strain in large volumes (Gavrilescu and Chisti, 2005; Venkata Mohan and Venkateswar Reddy, 2013). In this way, a variety of functional genes are expressed in different strains and work separately to avoid interaction between gene expression, which strengthens the core characteristics of each microbial strain, reducing the metabolic load of each bacterial chassis and is suitable for completing multiple complex tasks at the same time (Song et al., 2014; Ma et al., 2019).

Among the bioproduction-oriented synthetic consortia that have been designed and characterized so far (reviewed by Diender et al., 2021; Mittermeier et al., 2022), the co-cultures involving a model photosynthetic bacterium *Synechococcus elongatus* are primary examples of CO_2_-utilizing microbial platforms. Since the foundational work performed a decade ago (Ducat et al., 2012), the concept has been progressively developed. Sucrose-secreting *S. elongatus* was used in co-culture to support the growth of the several accompanying heterotrophic species (*Bacillus subtilis*, *Saccharomyces cerevisiae*, or *Escherichia coli*; Hays et al., 2016); and used for the production of an industrially relevant bioplastic polyhydroxybutyrate (PHB) in conjunction with *Halomonas boliviensis* (Weiss et al., 2017). Another polyhydroxyalkanoates production study explored the coupling of sucrose exporting *S. elongatus* with *Pseudomonas putida* (Löwe et al., 2017). More recently, the possibility of light-driven 3-hydroxypropionic acid production by the co-culture system composed of *S. elongatus* and *E. coli* was demonstrated (Hays et al., 2016). The same pair of organisms with different genetic modifications has been recently used to produce isoprene (Liu et al., 2021). All these studies shared the idea of designing the synthetic co-culture system capable of converting the photosynthetically produced sugar into high-value target molecules of biotechnological importance (Weiss et al., 2017). Furthermore, the stability of light-driven co-cultures can be improved by using hydrogel systems to ensure the space-efficient structure of the consortium (Gao et al., 2022). As the consortia-related methodological toolbox continues to expand (Ma et al., 2022; Singh and Ducat, 2022), the importance of the “phototroph + heterotroph” designs based on the conversion of CO_2_ to organic chemicals can be expected to grow.

**Figure 1:**
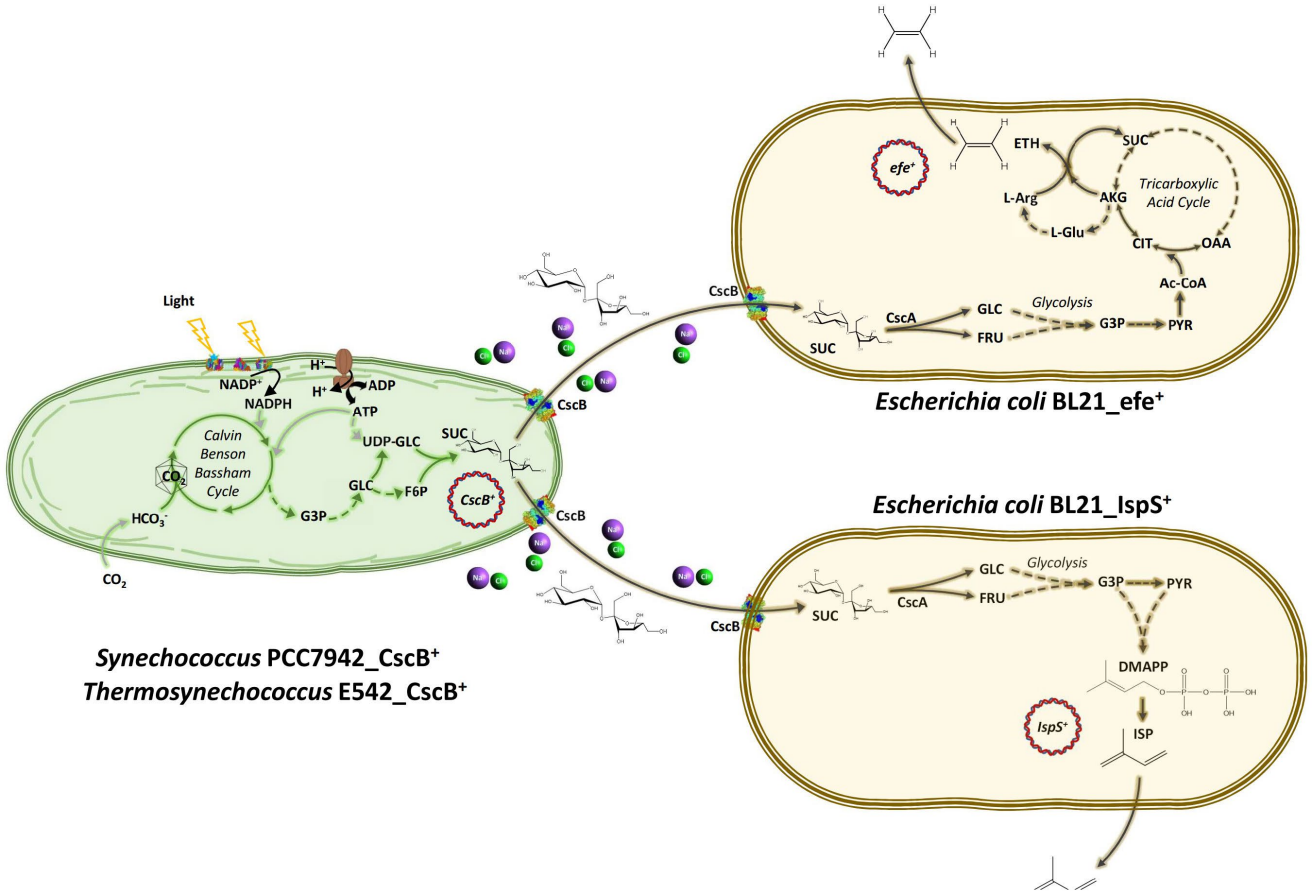
Schematic diagram of the artificial consortium system used in this study. *Thermosynechococcus* E542 and *Synechococcus* PCC7942 engineered to secrete sucrose under osmotic stress to support the growth of transgenic *Escherichia coli* strains capable of the synthesis of ethylene or isoprene. CO_2_, carbon dioxide; HCO_3_^−^, bicarbonate; G3P, glyceraldehyde 3-phosphate; GLC, glucose; FRU, fructose; PYR, pyruvate; UDP-Glc, uridine diphosphate glucose; F6P, fructose 6-phosphate; SUC, sucrose; Ac-CoA, acetyl CoA; CIT, citrate; AKG, alpha ketoglutarate; SUC, succinate; OAA, oxaloacetate; L-Glu, glutamate; L-Arg, L-Arginine; ETH, ethylene; DMAPP, dimethylallyl pyrophosphate; ISP, isoprene; ATP, adenosine triphosphate; ADP, adenosine diphosphate; NADP^+^, oxidized nicotinamide adenine dinucleotide phosphate; NADPH, reduced nicotinamide adenine dinucleotide phosphate; CscB, sucrose permease; CscA, sucrose hydrolase; IspS, isoprene synthase; and efe, ethylene forming enzyme.

Cyanobacteria are photosynthetic autotrophs with higher carbon fixation efficiency than plants (Wang et al., 2008). With a relatively simple genetic background, easy cultivation, and fast growth rates (Cheali et al., 2016), they are suitable microbial cell factories that avoid competition for land with food cultivation (Quintana et al., 2011). With the discovery of natural transformation (Kondo et al., 1994), electrotransformation (Thiel and Poo, 1989), and conjugation (Thiel and Peter Wolk, 1987) methods of plasmid uptake by the cyanobacterial cells, the existing barriers to importing target genes to cyanobacteria were eliminated, and the research on transgenic cyanobacteria is gradually increased. At present, research on cyanobacterial cell factories has covered many fields, such as the synthesis of high-value products and renewable compounds, carbon or nitrogen fixation, etc. (Choi et al., 2020; Tarawat et al., 2020; Mukherjee et al., 2021; Watanabe and Horiike, 2021). Cyanobacteria can synthesize sucrose, storage, and osmo-protective compound using triose phosphates, ATP, and electron equivalents generated during photosynthesis and native enzymes, including sucrose synthase (SPS; Huber and Huber, 1992). Although sucrose is not as common carbon source for *E. coli* as glucose, it has been found that about 50% of wild-type *E. coli* can utilize it (Holt et al., 1994). Under low carbohydrate conditions, the chromosomally coded sucrose catabolism genes—*csc,* namely *cscA*, *cscB,* and *cscK* are expressed in many *E. coli* strains K-12, BL21, O157:H7, and EC3132 (Hayashi et al., 2001; Jahreis et al., 2002). The imported sucrose is further broken down to glucose and fructose using CscA and metabolized as carbon and energy sources through glycolysis (Figure 1). Sucrose permease, sucrose: proton transporter encoded by the *cscB* gene, is a large proton symport system with 12 transmembrane helixes and three overlapping substrate specificities (Bockmann et al., 1992; Figure 1). It belongs to the oligosaccharide/H^+^ cotransporter subfamily in the Major Facilitator Superfamily (MFS; Marger and Saier, 1993), similar to LacY, and both can catalyze the oligosaccharide/H^+^ cotransport across the plasma membrane. Substrate specificity of CscB is to the fructofuranosyl ring of sucrose allows for sucrose, fructose, and lactulose, but not glucopyranosyl moiety to be metabolized (Sugihara et al., 2011). This makes sucrose a good intermediate for carbon and energy transfer between different members of an artificial community.

Some microorganisms can synthesize and secrete sucrose as an osmolyte when cultivated in excessive concentrations of salts (Hagemann, 2011). Exploration of this phenomenon in cyanobacteria enables continuous production of sucrose that exceeds the productivity of sugarcane, sugar beet, and other traditional sugar crops (Han and Watson, 1992). On this basis, cyanobacteria can be engineered to express sucrose permease, *CscB*, to secrete the disaccharide to the growth medium, and support the growth of *E. coli* in the absence of other carbon sources (Hays et al., 2016). For example, the sucrose yield of *Synechocystis* sp. PCC 6803 (3.13 mg/L/h) under 600 mM NaCl stress can be achieved by overexpressing genes related to sucrose synthesis and downstream sucrose metabolism (*sps*, *spp*, and *ugp* genes; Du et al., 2013). Meanwhile, *E. coli cscB* gene expression in *Synechococcus elongatus* PCC 7942 resulted in a yield of 36.1 mg/L/h (Ducat et al., 2012). Zhang et al. (2020) pointed out that sucrose derived from cyanobacteria may be considered an attractive renewable feedstock to produce chemicals. However, its separation and purification generate considerable costs. These costs can be eliminated by employing the microbial consortium involving a cyanobacterium that provides photosynthetically produced sucrose to *E. coli*, which in turn serves as a producer of the target chemical.

In this study, artificial consortia consisting of cyanobacteria: *Thermosynechococcus elongatus* PKUAC-SCTE542 (subsequently, E542), *Synechococcus elongatus* PCC7942 (subsequently, PCC7942) in conjunction with *E. coli* BL21 expressing either IspS or Efe were constructed to produce volatile platform hydrocarbons indirectly from CO_2_. In this system, cyanobacteria overexpressing the *E. coli cscB* gene under the control of a light-driven promoter produced and exported sucrose into the medium. The second component of the consortium, *E. coli* engineered with ethylene-forming enzyme gene *efe*, or isoprene synthetase gene *ispS*, can use sucrose in the medium and produce volatile platform hydrocarbons.

## Materials and methods

### Plasmid construction

All the strains used in this study are listed in Table 1. All plasmids (Supplementary Table 1) were created using oligonucleotides summarized in Supplementary Table 2. The polymerase chain reaction was performed using Phanta Max 2x (Vazyme, China) according to the manufacturer’s recommendations. Constructs were assembled using ligase-independent ClonExpress II One Step Cloning Kit (Vazyme, China) using the manufacturer’s guidelines, propagated in *E. coli* DH5α, and sequence verified by Sanger sequencing. The ethylene-forming enzyme (*efe*) of *Pseudomonas syringae pv. Phaseolicola* (AF101058.1) and isoprene synthetase (*ispS*) from *E. globulus* (BAF02831) were synthesized commercially at BGI WRITE (Beijing, China). After PCR amplification with their respective primers (Supplementary Table 2), both genes were separately cloned into the bacterial expression plasmid pBAD_LIC_cloning vector (8A) linearized with EcoRV essentially as described earlier (Cui et al., 2022; Cui and Daroch, 2022). The plasmids were propagated under 50 μg/ml ampicillin selection, and both enzymes were expressed under arabinose promoter (*araBAD*) with 0.2% arabinose.

**Table 1:** Strains used in the study.

Strains	Genotype	Source
DH5α	F^−^ *φ80 lacZ ΔM15 Δ(lacZYA-argF)U169 endA1 recA1 hsdR17 (r_k_^−^, m_k_^+^) supE44 λ^−^ thi-1 gyrA96 relA1 phoA*	From ktsm-life
BL21(DE3)	F^−^ *ompT hsdS(r_B_^−^m_B_^−^) gal dcm*(DE3)	From ktsm-life
Conjugation helper strain	*E. coli* HB101 harboring pRL443 and pRL623	Zhang et al. (2020)
*Thermosynechococcus elongatus* PKUAC-SCTE542	Thermophilic cyanobacteria collected from the hot springs in Ganzi area, Sichuan, China	Tang et al. (2018) and Liang et al. (2019)
*Synechococcus elongatus* PCC 7942	*Synechococcus elongatus* PCC 7942 wild type	PCC Collection

The self-replicative plasmid expressing *cscB* gene (pLJD31) was created using a five-way assembly of the following fragments. The backbone and antibiotic resistance gene promoters were amplified from pAM5057 using MDLJCSTR224 and MDLJCSTR225, and MDLJCSTR500 and MDLJCSTR501, respectively. Plasmid pAM5057 was a gift from Susan Golden (Addgene plasmid # 120085; http://n2t.net/addgene:120085; RRID:Addgene_120,085). The kanamycin resistance gene was amplified from pETM11_Saz_CA (Hou et al., 2019) based on pETM11 backbone (EMBL Heidelberg) using MDLJCSTR120 and MDLJCSTR161. Light-driven promoter PsbA* along with the RBSV33 of *Synechocystis* PCC6803 (Wang et al., 2018) were custom synthesized at BGI WRITE (Beijing, China) and amplified using primer pair MDLJCSTR502 and MDLJCSTR136. The *cscB* gene expressing sucrose permease was amplified from *E. coli* DH5α using MDLJCSTR226 and MDLJCSTR227. The resultant plasmid pLJD31 was used for the transformation of E542.

The integrative plasmid expressing *cscB* gene was created using a three-step procedure. First, the plasmid pLJD50 was constructed based on pAM2991 backbone targeting the Neutral Site I of PCC7942 genome. The backbone was amplified with MDLJCSTR290 and MDLJCSTR291 primer pair. The plasmid pAM2991 was a gift from Susan Golden (Addgene plasmid # 40248; http://n2t.net/addgene:40248; RRID:Addgene_40,248; Ivleva et al., 2005). The antibiotic resistance promoter P_amp_, spectinomycin resistance gene, the light-driven promoter of PCC7942, multiple cloning site, RBS, and terminator were amplified from pETS1 plasmid (Liang et al., 2019) using primer pair MDLJCSTR282 and MDLJCSTR228. Second, from pLJD50 vector a kanamycin-containing derivative pLJD51 was generated using a two-way assembly of PCR products amplified with primer pair MDLJCSTR207-MDLJCSTR289, and MDLJCSTR161-MDLJCSTR162. Finally, pLJD51 was linearized with MDLJCSTR319 and MDLJCSTR320 pair and assembled with *cscB* PCR product amplified with MDLJCSTR381 MDLJCSTR382 pair from the gDNA of *E. coli* DH5α. The resultant plasmid pLJD32 was used to transform PCC7942.

### Recombinant strains construction

The modified plasmids with *efe* (pBAD-*efe*) or *IspS* (pBAD-*IspS*) were transformed to *E. coli* BL21 to produce ethylene or isoprene. The *cscB* containing plasmid (pLJD31) was transformed into E542 through the conjugation *via E. coli* HB101 harboring pRL443 and pRL623 (Thiel and Peter Wolk, 1987) and *E. coli* DH5α carrying the pLJD31 plasmids, respectively. Meanwhile, the pLJD32 was transformed into PCC7942 using the recently optimized protocol (Riaz et al., 2022).

### Culture conditions

Two co-culture media, Co-BG11 and M9-BG11, were tested to establish synthetic consortia between *E. coli* BL21 and E542 and PCC 7942. The Co-BG11 medium was prepared as described earlier (Zhang et al., 2020). In short, the medium was based on a conventional BG-11 medium and modified with the supplementation of 150 mM NaCl, 4 mM NH_4_Cl, and 3 g/L 2-[[1,3-dihydroxy-2-(hydroxymethyl) propan-2-yl] amino] ethanesulfonic acid (TES) to support the growth of *E. coli*. In addition, NaCl was also added to induce stress in cyanobacterial strains for sucrose production and secretion. The second co-culture medium, M9-BG11 was designed based on regular BG-11 and M9 minimal media optimal for cyanobacteria and *E. coli*. The original BG11 medium (Stanier et al., 1971) was supplemented with 6 g/L K_2_HPO_4_, 3 g/L KH_2_PO_4_, 150 mM NaCl, 1 g/L NH_4_Cl, 0.12 g/L MgSO_4_, 0.014 g/L CaCl_2_, and 0.001 g/L vitamin B1. In the consortia of ethylene, 0.2 g/l FeSO_4_ was supplemented.

For cloning and precultures, *E. coli* cells were routinely grown in liquid LB medium in a shaking incubator (HZQ-X300C, Yiheng, Shanghai, China) at 180 rpm or LB agar (solid) plates at 37°C. Further, 50 μg/ml of ampicillin was added to maintain plasmids, and 0.2% arabinose was used to induce the expression of *efe* and *IspS* genes.

Cyanobacteria were cultivated in BG11 medium in an illuminated shaking incubator (Jintan Jingda Instruments TS-2112B) under constant illumination with white light at 2000 LUX (~36 μmol m^−2^ s^−1^) at 180 rpm at 37°C for PCC7942 and 45°C for E542. In addition, the medium was supplemented with 50 μg/ml of kanamycin to maintain the plasmids and genomic integration.

Before establishing synthetic consortia, cyanobacteria grown in the exponential phase were diluted into one of the two co-culture media (Co-BG11 and M9-BG11) at a cell density of OD_750_ ≈ 0.15 (corresponding to 9 × 10^6^ cells/ml) and grown at 37°C (PCC7942) or 45°C (E542) for 48 h to reach the cell density of OD_730_ of 0.3. The *E. coli* BL21 cells were preincubated in the M9 medium supplemented with antibiotic and 2 g/L sucrose instead of glucose for 12 h. Later, the cells were collected by centrifugation, washed twice with deionized water, and inoculated into the 15 ml cyanobacterial culture described above to the final cell density of OD_600_ = 0.1–0.2 (corresponding to 1 × 10^7^–2 × 10^7^ cells/ml). After adding arabinose to the concentration of 0.2%, the co-culture was incubated at 30°C for 48 h to produce ethylene or isoprene.

### Quantification of cells

For pure cultures of cyanobacteria and *E. coli*, cell density was measured at OD_730_ and OD_600_ with an EPOCH microplate reader (BIOTEK, United States). For the co-culture, the cell number of *E. coli* was calculated by counting colony-forming units (CFU) in LB plate after 14 h of incubation at 37°C, and the cell number of cyanobacteria was counted by Countstar-IC1000 Cell Analyzer (Ruiyu, China).

### Real-time RT-PCR analysis

Approximately 1.5–2 ml *E. coli* culture at OD_600_ = 0.4–0.6 was collected by centrifugation at 4°C, 8,000 rpm for 3 min to isolate RNA with Total RNA kit I (Omega Bio-Tec, United States) to compare the mRNA levels of sucrose catabolism genes (*cscB*, *cscA*, and *cscK*) and *efe* or *IspS* gene in M9 media supplemented with 2 g/L sucrose or conventional LB media. The reference gene for *E. coli* RT-qPCR is 16S rRNA. In the case of cyanobacteria, approximately 50–100 ml cultured cells were centrifugated at 4°C 8,000 rpm for 15 min to isolate total RNA to verify the mRNA levels of *cscB* gene in engineered strains. The reference gene for E542 is an “analysis fragment” from the literature at positions “2,985–3,144” of CP032152, and *purl* was used for PCC7942 (Riaz et al., 2021). The total RNA of *E. coli* was isolated with Total RNA kit I (Omega Bio-Tech, United States), while the total RNA of cyanobacterial was isolated with Trizol Reagent (Invitrogen, United States; Chomczynski and Sacchi, 2006). PrimeScript™ RT reagent Kit with gDNA Eraser (Takara, Japan) was used to remove the gDNA and reverse transcribe RNA to cDNA. The RT-qPCR reaction mix was prepared with TB Green Premix Ex Taq II (TaKaRa) in a QuantStudio 5 real-time system (ABI, United States). All the primers used for RT-qPCR analysis are given in Supplementary Table 2.

### Quantification of ethylene production

The ethylene production in the gas phase was performed in a 60 ml sealed bottle headspace with 15 ml cyanobacterial-*E. coli* co-culture essentially as described before (Cui et al., 2022). In short, 1 ml air sample was injected into Agilent Technology 6850 GC FID with Porapak Q 3 M × 1/8 column to measure the concentration of ethylene. Three biological replicates were prepared to measure ethylene production, while one was maintained to determine the cellular density (CFU or OD). Ethylene production was defined as the amount (1 μmol) of ethylene produced by 10^11^ cells. The ethylene production per day was calculated as follows:


$$\mathrm{Ethylene production}=\frac{C\cdot Va}{22.4\;\mathrm{\mu L}/\mu \mathrm{mol}\cdot 1,000\cdot N\cdot T\;}$$

*C*: concentration of ethylene in the bottle, measured by GC (ppm); *V*a: gas volume in the bottle (mL); *N*: cell number; and *T*: reaction time (day).

### Quantification of isoprene production

Isoprene production in the gas phase was analyzed in a headspace of a 60 ml sealed bottle with 15 ml co-cultured cells, as described before (Cui and Daroch, 2022). Briefly, toluene was injected into the bottle to absorb isoprene and incubated overnight at 4°C to condense the isoprene. The Agilent Technology 6850 N-5975 GC–MS with HP-5MS 5% Phenyl Methyl Siloxa column was used to measure the isoprene concentration. Different gradient volumes of liquid isoprene were added to the bottle and detected with GC–MS to make the standard curve. Three replicas for each strain were prepared to measure isoprene, while one replica was used for the cell density measurement (CFU or OD). The efficiency of isoprene production was calculated as follows:


$$\mathrm{Isoprene production}=\frac{W}{68.11\frac{\mathrm{\mu g}}{\mu \mathrm{mol}}\cdot N\cdot T}$$

*W*: the content of isoprene in the bottle (μg); *N*: cell number; and *T*: reaction time (day).

### Quantification of extracellular sucrose content

The 20 ml cyanobacterial culture supernatants grown under NaCl stress were collected and concentrated ten times to 2 ml at 65°C. The concentrated sucrose solution was hydrolyzed with 2 ml 6 mol/l HCl for 10 min at 100°C, then pH was adjusted to 9, and ddH_2_O was added to the final volume of 10 ml. Subsequently, 0.8 ml of this liquid was mixed with 0.6 ml DNS detection reagent (Casmart, China) and boiled for 5 min at 100°C. The sucrose concentration was determined at OD_520_ against the standard curve prepared for known concentrations of sucrose treated under the same conditions (Miller, 1959).

## Results

### Growth and sucrose secretion of cyanobacterial cells

#### Growth parameters and gene expression analysis of sucrose-producing cyanobacteria

E542 and PCC7942 were engineered to secrete sucrose by expressing the sucrose permease encoding gene *cscB* under the light-driven promoters (Supplementary Figure 1) and the kanamycin resistance gene. The plasmids were transferred into the cyanobacteria E542 and PCC7942 using conjugation and natural transformation, respectively. The insertion of *cscB* gene and sucrose secretion did not significantly affect the growth of cyanobacteria under their optimal growth conditions (Supplementary Figure 2). As is shown in RT-qPCR results (Table 2), the *cscB* gene was successfully transcribed in engineered strains. The mRNA expression levels of the cells grown under constant illumination were 8.01 and 7.22 fold higher than the reference for PCC7942 and E542, respectively.

**Table 2:** Relative gene expression levels of *cscB* in engineered strains.

	delta–delta Ct value (∆∆Ct)				Fold change (2^–∆∆Ct^)
	1	2	3	Average	
PCC7942_*cscB^+^*	2.36	3.60	3.04	3.00	8.01
E542_ *cscB^+^*	2.00	3.63	2.93	2.85	7.22

#### Sucrose secretion of engineered strains

**Figure 2:**
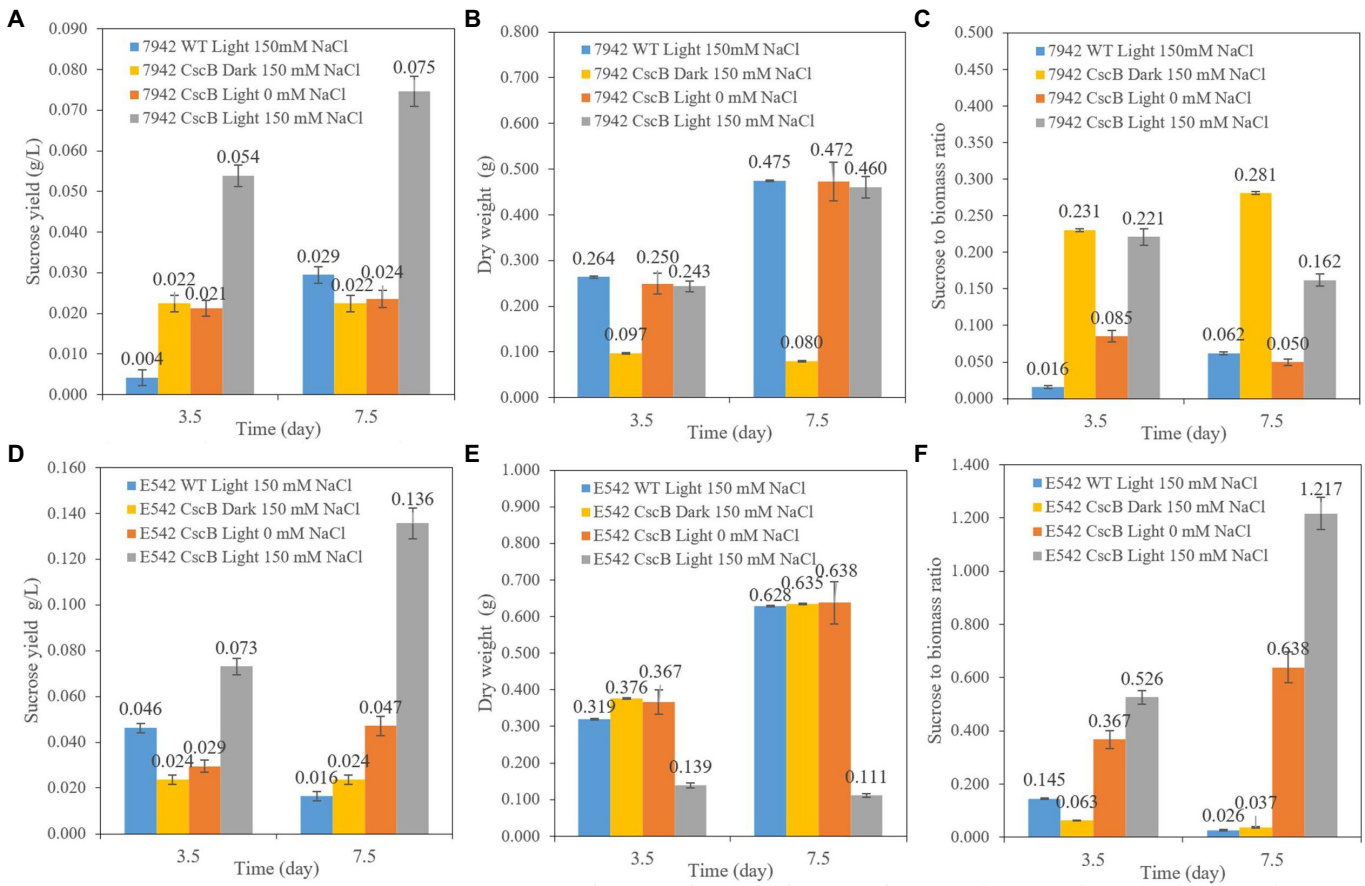
The comparison of sucrose yield of the PCC7942_*cscB^+^* **(A)**, E542*_cscB^+^* **(D)**, accumulated dry weight **(B,E)**, and sucrose to biomass ratio **(C,F)** grown under different light and osmotic pressure in BG11 growth medium. Height of the bars shows the mean of three independent experiments with error bars representing SD from measurements.

The cells of E542_*cscB^+^* and PCC7942_ *cscB^+^* were cultured from an initial OD_730_ = 0.1–0.13 at 45°C (E542) or 37°C (PCC7942). Their growth and sucrose secretion was monitored for 3.5 and 7.5 days, as shown in Figure 2. Compared with the wild-type strains (WT), the sucrose yield of the engineered strains increased when cultivated under the same culture conditions. Compared with the yield in the dark environment and in the culture environment without NaCl, the sucrose yield of the engineered strains significantly increased under 150 mM NaCl with light. The sucrose yield in E542*_cscB^+^* reached 0.073 g/L (0.234 g/L/OD_730_) and 0.136 g/L (0.221 g/L/OD_730_) after 3.5 and 7.5 days, respectively, and the secretion efficiency remained at about 0.02 g/L/day. However, sucrose yield in PCC7942*_cscB^+^* was 0.054 g/L (0.226 g/L/OD_730_) and 0.075 g/L (0.166 g/L/OD_730_) after 3.5 and 7.5 days, respectively. The yield decreased slightly under long-term culture, and the maximum secretion efficiency was about 0.015 g/L/day. The secretion efficiency is lower than the recent study with *Synechococcus elongatus* UTEX 2973 cultured in BG11 + 150 mM NaCl (~0.1 g/L/day, ~0.59 g/L/OD_730_ for 3 days; Zhang et al., 2020) and the other studies with PCC7942 and *lac* promoter in other media (0.05–0.3 g/L/day for 2–4 days, total production is 0.156–0.625 g/L, 0.07–0.25 g/L/OD_730_; Hays et al., 2016; Li et al., 2017). The carbon allocation ratio analysis shows an interesting difference between both transgenic cyanobacteria. Examination of the PCC7942_ *cscB^+^* indicates that sucrose secretion at dark remains relatively stable, which, combined with low biomass production, yields relatively high carbon partitioning to sucrose (Figures 2A–C). A different effect is observed in E542_*cscB^+^* where the dry weight upon transfer to dark conditions remains stable and sucrose secretion almost halts, resulting in very low sucrose to biomass ratio. Conversely, prolonged incubation at saline illuminated conditions results in low biomass accumulation and maximal sucrose productivity and carbon partitioning to sucrose significantly exceeding 1, indicating the majority of the flux being directed toward sucrose secretion. Similar results have been obtained before (Ducat et al., 2012), indicating that a high stress level is required to maximize sucrose production.

Both the strain and the constructs may influence the sucrose production of the transgenic cells and the sucrose secretion efficiency. Besides, cell density may be a factor affecting sucrose secretion yield. If the sucrose secretion efficiency of single cells is the same, a lower growth rate and cell density may lead to a lower sucrose yield per liter cell culture.

**Figure 3:**
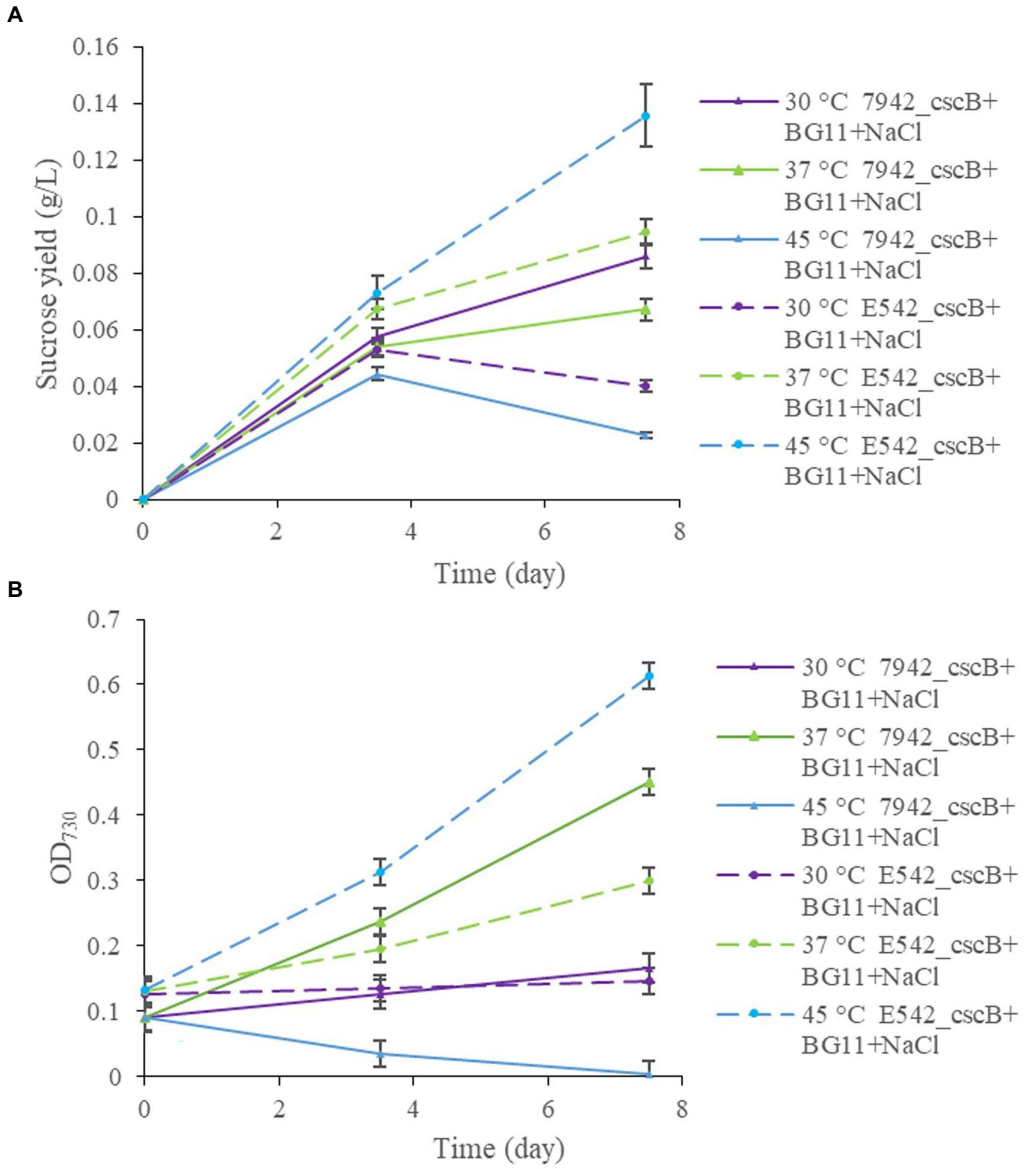
The comparison of sucrose yield **(A)**, and growth curve **(B)**, of engineered E542*_cscB^+^* and 7,942*_cscB^+^* strains at different temperatures grown in BG-11 medium under salt stress. Distribution of the data points shows the mean of three independent experiments with error bars representing SD from measurements.

The two engineered strains were cultured at different temperatures (30, 37, and 45°C), and samples were taken at 3.5 and 7.5 days to measure and compare the sucrose content in the culture medium and the growth of the strains (Figure 3). At 30°C, E542*_cscB^+^* grows slowly (Figure 3B), and the sucrose yield (0.053 g/L for 3.5 days; 0.040 g/L for 7.5 days; Figure 3A) was also lower, while the sucrose yield of PCC7942*_cscB^+^* (0.057 g/L for 3.5 days; 0.085 g/L for 7.5 days) was better at this temperature. At 37°C, both strains could grow well, and the 0.094 g/L sucrose yield of E542*_cscB^+^* for 7.5 days was better than 0.067 g/L of PCC7942*_cscB^+^*. At 45°C, the E542 grew well, and the sucrose yield was the highest (0.136 g/L for 7.5 days), while PCC7942 died gradually due to poor tolerance to high temperature. Consequently, no sucrose was secreted by PCC7942*_cscB^+^* at 7.5 days. The changes in sucrose yield were consistent with the changes in cell density during this process. Sucrose yield was higher when cells grew well and lowered at lower growth rates. Therefore, the temperature significantly affected the sucrose secretion by the engineered strains by impacting their sucrose biosynthesis, growth, or both. Since sucrose biosynthesis in cyanobacteria strains was not artificially regulated in this experiment, the biosynthesis mainly came from the overflow of the photosynthate produced, and the efficiency may be biased toward strains’ respective optimal growth temperatures (Warr et al., 1985). Longer cultivation of the co-culture exceeding 10 days resulted in the cell growth approaching a plateau, probably due to decreased cell viability and saturation of sucrose secretion capacity of the community.

### Growth of BL21_*efe^+^* and BL21_*IspS^+^* in the co-culture medium and gene expression

#### Growth of *Escherichia coli* strains in the co-culture medium

To ensure the suitability of selected co-culture growth media for the cultivation of engineered *E. coli* strains and sucrose uptake; the two transgenic strains were cultured in derivatives of sucrose-containing media to choose the optimal composition for co-culture studies. Previous studies showed that *E. coli* ATCC#9637 *ΔcscR* strain required about 1.2 g/L sucrose to grow well (that varied depending on other elements of the growth medium; Hays et al., 2016), while the sucrose yield achieved by the transgenic cyanobacteria typically does not reach this level. Therefore, in the preliminary experiment designed to determine the initial cultivation conditions, the co-culture environment was simulated by adding sucrose to the final concentration of 0.2 and 2 g/L in both of the co-culture media, i.e., co-BG11 and M9-BG11. The experiment was performed at 30 and 37°C (Supplementary Figure 3). Results showed the good growth of engineered *E. coli* BL21_*Efe_PS* at 37°C when 2 g/L sucrose was added to each of the co-culture media, while the growth at 30°C was much poorer, with the final cell density of OD_600_ = 0.4, markedly lower than other conditions tested (Supplementary Figure 3). This may be due to the high energy consumption of the cells at low temperature, and sucrose being gradually consumed during 96 h of cultivation and insufficient to provide sufficient carbon and energy for the rapid proliferation of *E. coli*. The lower concentration (0.2 g/L) of sucrose in the medium significantly inhibited the growth of *E. coli*.

#### Gene expression of *Escherichia coli* BL21_efe^+^ and BL21_IspS^+^ in sucrose-containing medium

**Figure 4:**
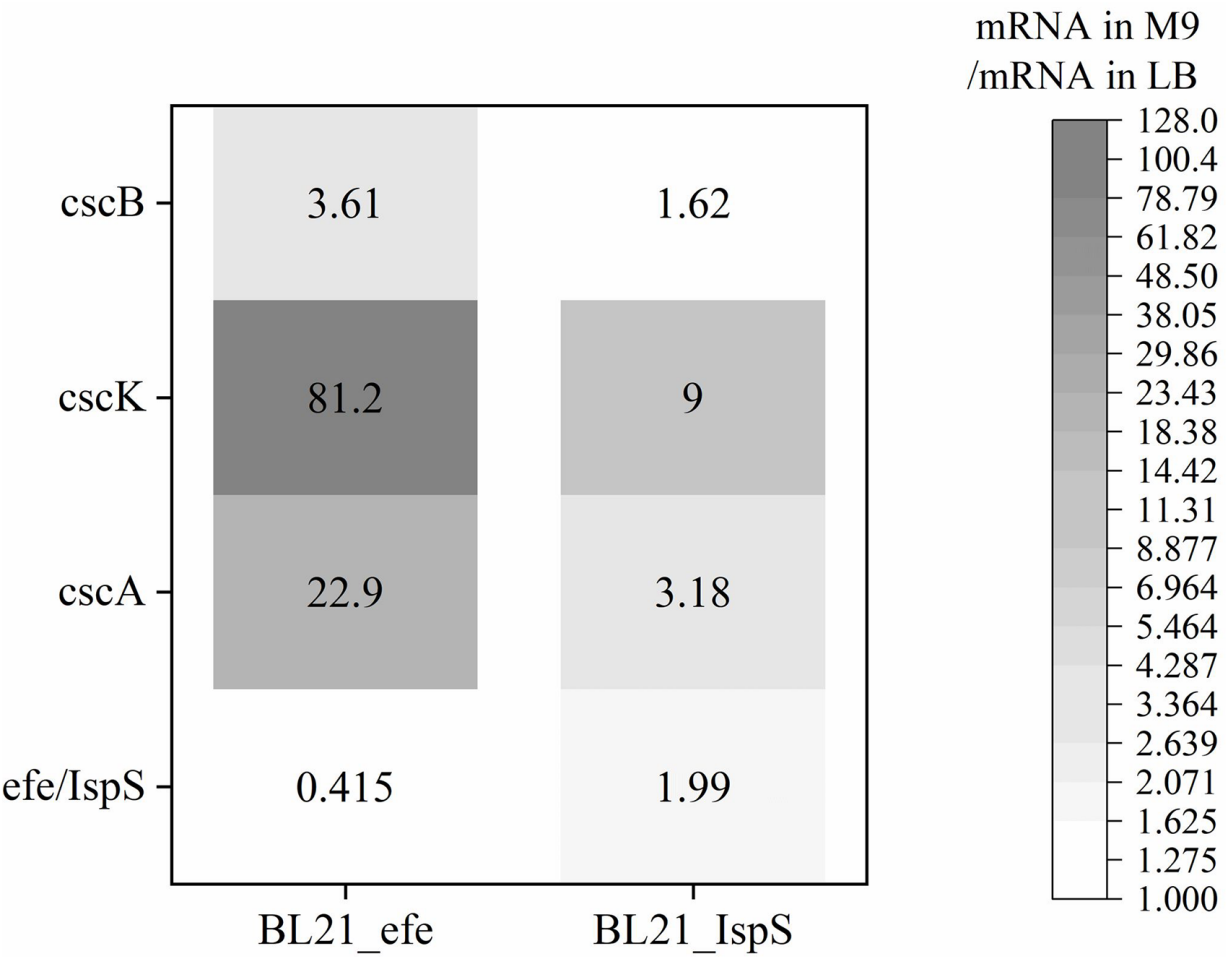
Changes of gene expression of BL21_*efe-PS* and BL21_*IspS_EG^+^* in the cultures with sucrose as the sole carbon source calculated using the relevant ratio of the mRNA value in the M9 medium to the mRNA value in the LB with 2^−ΔΔCT^ method (Livak and Schmittgen, 2001).

The expression of sucrose metabolism-related genes (*cscA*, *cscB*, and *cscK*) and the volatile platform hydrocarbon synthase genes (*efe* and *IspS*) in engineered *E. coli* strains cultured with sucrose as the sole carbon source was studied. After BL21_*efe_PS^+^* and BL21_*IspS_EG^+^* strains were cultured to OD_600_ = 0.4–0.6 in LB and M9 medium (glucose in M9 medium was replaced with 2 g/L sucrose), 0.2% arabinose was added to induce the expression. After induction at 30°C for 5 h, total RNA was extracted and detected. mRNA levels of the two strains in the M9 medium containing sucrose were compared with those in the LB medium (Figure 4). As shown in Figure 4, the RNA transcription levels of sucrose hydrolase gene *cscA* and fructokinase gene *cscK* increased significantly in the BL21_*efe^+^* and BL21_*IspS^+^*, while the increase for sucrose permease gene *cscB* was not significant, indicating sufficient sucrose flux. In the two strains, the transcription of all *csc* genes in BL21_*IspS_EG^+^* was significantly lower than that of BL21_*efe_PS^+^*. This may be related to the different synthesis pathways of their precursors in *E. coli* (Figure 1). In *E. coli*, the main precursors of ethylene synthesis are α-ketoglutaric acid and arginine. Arginine is an amino acid, which can be obtained directly from tryptone in LB medium, while α-ketoglutaric acid is synthesized by the TCA cycle (Li et al., 2006; Xiao et al., 2016). Acetyl-CoA, an essential node in the TCA cycle, can be derived from glycolysis and the decomposition of fatty acids and amino acids; meanwhile, it is also a building block for synthesizing these compounds (Han et al., 2001; Luo et al., 2014). In LB medium, raw materials such as amino acids are abundant, but the carbohydrate content is low, so presumably lower portion of acetyl-CoA is synthesized from glycolysis. However, in an M9 medium supplemented with sucrose, amino acids and other building blocks are likely to be limiting. Presumably, the resultant metabolism depends primarily on glycolysis to synthesize acetyl-CoA and, ultimately α-ketoglutaric acid, amino acids, and other essential substances to keep the cells alive. Therefore, the transcription and expression of related genes (*csc* genes) were likely to be increased under the increased metabolic requirements of glycolysis. However, since the synthesis of α -ketoglutaric acid was lower, the supply of arginine was limited; consequently, the ethylene synthesis could also be limited by the availability of precursors. On the contrary, the precursors of isoprene synthesis in this *E. coli* strain are pyruvate and glyceraldehyde-3-phosphate, both directly linked to glycolysis, and *csc* series genes may have been expressed in LB medium. Compared with the LB medium, precursors can be directly synthesized from sucrose available in the M9 medium, and the increase in precursor supply was conducive to the synthesis of isoprene and promoted the transcription of related genes.

#### Growth of engineered cyanobacteria in co-culture media

**Figure 5:**
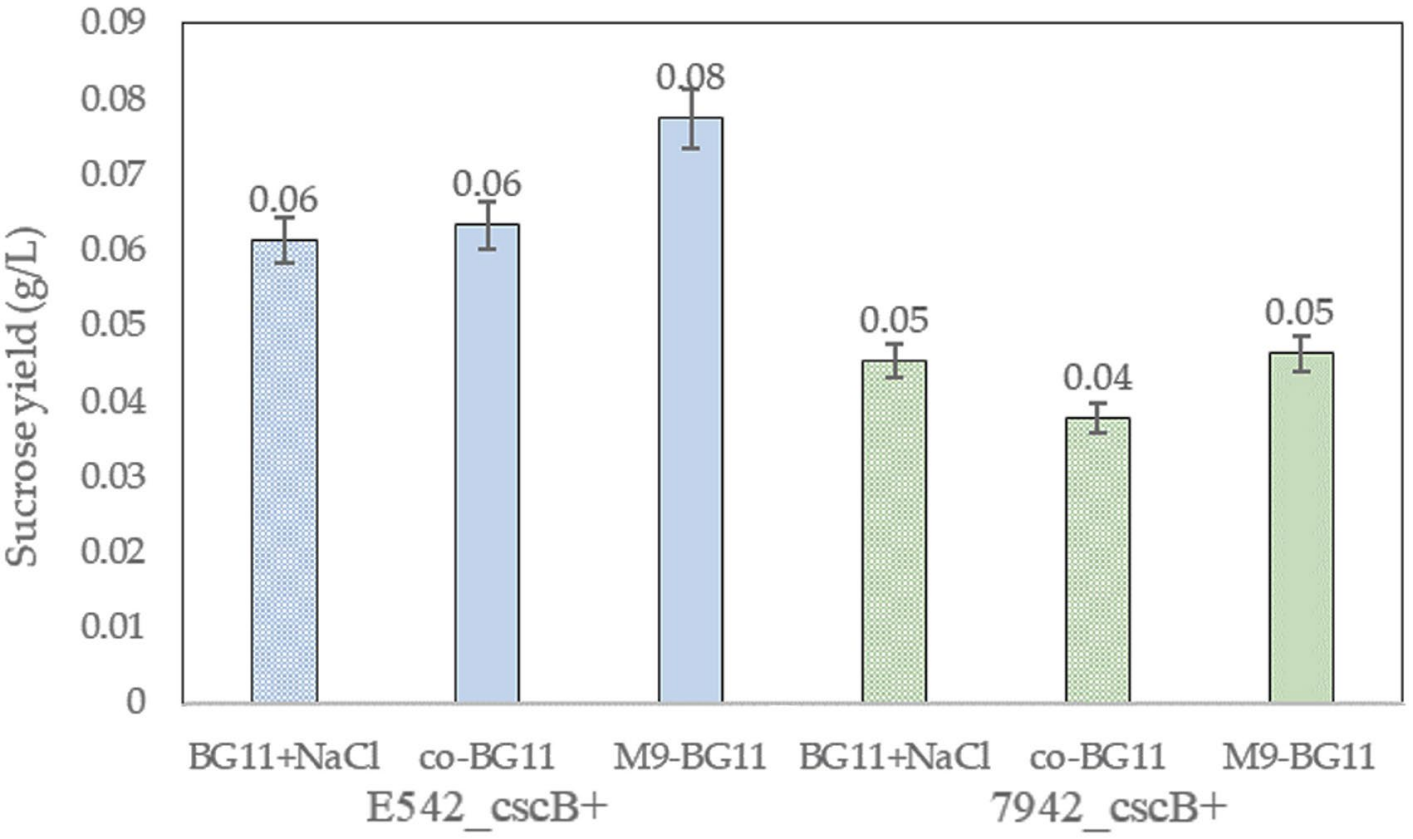
The comparison of sucrose yield of the engineered strains of E542*_cscB^+^* and PCC7942_*cscB^+^* in different co-culture media during 48-h long cultivation. Height of the bars shows the mean of three independent experiments with error bars representing SD from measurements.

The addition of arabinose and extra ions to the co-culture media may affect the growth of cyanobacteria. E542_*cscB^+^* and PCC7942_*cscB^+^* were cultured in BG11 and the two co-culture media to explore these effects. Cyanobacteria can grow normally in both co-culture media, but the growth in the co-BG11 medium was somewhat slower (Supplementary Figure 4). Meanwhile, the addition of 0.2% arabinose had no significant effect on the growth of the strains. The sucrose yield in different media was tested to explore the sucrose secretion capacity of engineered strains in the co-culture system (Figure 5). The culture was started with OD_730_ = 0.15 and the sucrose yield of the strains cultured at their optimal growth temperatures for 2 days was detected. As shown in Figure 5, the sucrose yield of E542_*cscB^+^* was higher than that of PCC7942*_cscB^+^* in all media; this was consistent with the prior results of measurement for sucrose secretion by other transgenic cyanobacteria. Both the OD_730_ and sucrose yield in the M9-BG11 medium was higher than that of the co-BG11 medium (Figure 5; Supplementary Figure 4), indicating its more optimal composition for this task.

### Production of volatile platform hydrocarbons in artificial consortium systems

Based on the literature and previous studies (Cui et al., 2022; Cui and Daroch, 2022), ethylene synthase and isoprene synthase were expressed well when the induced temperature was lower than the growth temperature of *E. coli* (37°C), and both proteins were prone to inclusion body formation at high temperature. In analyzing sucrose secretion by cyanobacteria above, engineered PCC7942 performed better at 30 and 37°C. Meanwhile, E542 secreted little sucrose at 30°C due to the poor growth parameters of the strain at this temperature. To compensate for those drawbacks, sucrose secretion during co-culture could be enhanced by increasing initial cell density. Based on the productivity data per cell, these lower growth rates may have better channeled the available cellular resources for sucrose production. Therefore, 30°C was selected as the reaction condition for co-cultures. Before that, cells were cultured separately to maintain good initial activity and density.

The artificial consortium systems of engineered strains of cyanobacteria E542*_cscB^+^* and PCC7942*_cscB^+^* with engineered strains of *E. coli* BL21_*efe_PS^+^* were established at co-BG11 medium and M9-BG11 media. After culturing for 48 h at 30°C, ethylene was detected by the GC. At the same time, the number of *E. coli* cells in the co-culture system was calculated by the CFU. The results of ethylene productivity are shown in Table 3. *Escherichia coli* BL21_*efe_PS^+^* was cultured in the co-culture medium supplemented with 2 g/L sucrose without cyanobacteria as the control group.

**Table 3:** Ethylene and isoprene production in consortium system.

	Ethylene production			Isoprene production		
	Production (μmol/L)	Production (μmol/10^11^ cells)	*V* (μmol/10^11^ cells/day)	Production (μmol/L)	Production (μmol/10^11^ cells)	*V* (μmol/10^11^ cells/day)
PCC7942 Co-BG11	16.94 ± 1.80	112.97	56.48	TLTD	/	/
PCC7942 M9-BG11	43.82 ± 8.42	219.08	109.54	0.75 ± 0.05	3.74	1.87
E542 Co- BG11	10.60 ± 1.96	70.64	35.32	TLTD	/	/
E542 M9-BG11	52.71 ± 0.18	263.55	131.77	0.39 ± 0.01	1.94	0.97
Co-BG11 + 0.05 g/L sucrose (only *E. coli*)	17.81 ± 1.85	59.37	29.69	TLTD	/	/
M9-BG11 + 0.05 g/L sucrose (only *E. coli*)	25.78 ± 0.96	64.47	32.23	0.34 ± 0.06	1.66	0.83
Co-BG11 + 2 g/L sucrose (only *E. coli*)	73.93 ± 5.26	164.30	82.15	0.38 ± 0.05	1.25	0.64
M9-BG11 + 2 g/L sucrose (only *E. coli*)	89.16 ± 2.98	139.31	69.65	1.67 ± 0.10	2.57	1.29
LB (Cui et al., 2022; Cui and Daroch, 2022)	794.07 ± 23.82	2908.7	13961.8	0.97 ± 0.09	0.97	1.94

TLTD, Too low to detect (below 0.2 μmol/L).

During the co-culture process, *E. coli* cell density was stable at the level of 1.5 × 10^7^–2 × 10^7^/ml and cyanobacteria grew normally in the system. The ratio of cyanobacteria and *E coli* cells was from 8.3 (15 × 10^7^ cyanobacteria per milliliter/1.8 × 10^7^ *E.coli* per milliliter, at the beginning) to 10.6 (20 × 10^7^ vs. 1.89 × 10^7^, after 1 day) then 12.5 (25 × 10^7^ vs. 2.0 × 10^7^, after 2 days) in consortium with M9-BG11. While the ratio was from 8.2 (15 × 10^7^ cyanobacteria per milliliter/1.83 × 10^7^ *E.coli* per milliliter, at the beginning) to 10.2 (19 × 10^7^ vs. 1.86 × 10^7^, after 1 day), and then 11.5 (22 × 10^7^ vs. 1.92 × 10^7^, after 2 days) in the consortium with Co-BG11. No other species was observed under microscopy.

The ethylene production was observed in all consortia, but the ethylene production was lower than that of the individual *E. coli* strain grown in LB (794.07 μmol/L for 5 h), or in the two co-culture media with 2 g/L sucrose (70.93 and 89.16 μmol/L for 48 h). In the studies that utilized *E. coli* as a single producer, the ethylene production level was 6–500 μmol/L/h in the strains with different vectors (Digiacomo et al., 2014; Eckert et al., 2014). In PCC7942, the productivity of ~20 μmol/L/h was recorded with psbAI promoter (Takahama et al., 2003) and ~ 6.25 μmol/L/h was recorded with *trc* promoter (Carbonell et al., 2019), while in the engineered strain of *Synechocystis* PCC 6803 the levels of 12–42 μmol/L/h were observed (Lee et al., 2015; Veetil et al., 2017; Wang et al., 2018). The comparison shows that the E542 strain and M9-BG11 medium combination had the highest ethylene yield, 52.71 μmol/L culture for 2 days, among the conditions tested. Combined with sucrose secretion and the growth of *E. coli*, inadequate nutrition leading to low cell density and low metabolic efficiency may be the reason for sub-par productivity. It is worth mentioning here that the approach of submerged cultivation is not the only one reported for ethylene bioproduction. A system based on cyanobacterial biofilms was designed to achieve a 2.2-fold increase in the ethylene production yield compared to the cell suspension (Vajravel et al., 2020).

The consortium systems of cyanobacteria and *E. coli* for isoprene production were constructed in the same media and conditions as for ethylene production. Isoprene concentration was detected by GC after culturing for 48 h. At the same time, the number of *E. coli* cells in the co-culture system was calculated by CFU. The results of isoprene productivity are shown in Table 3. In three instances, the isoprene productivity of the consortium was lower than the sensitivity of the assay, i.e., 0.2 μmol/L. The control group of *E. coli* BL21_IspS_EG^+^ was cultured in the co-culture medium supplemented with 2 g/L sucrose without cyanobacteria.

The change in cell ratio was similar in the ethylene co-culture system. The comparison shows that the PCC7942 strain and M9-BG11 medium combination had the highest isoprene yield, 0.75 μmol/L culture for 48 h, among the conditions tested. Compared with the production per liter of individual BL21_*IspS_EG^+^* cell culture under carbon saturation, the isoprene production per liter of cell culture in each co-culture system also decreased, but not significantly. Especially, isoprene production per cell was especially stable, suggesting that the decrease resulted from lower sucrose availability. The isoprene production of individual strains expressing *IspS* gene in the other studies was 0.4–1.25 mg/L *E. coli* for 18 h in M9 media (Zurbriggen et al., 2012) and 3–7 mg/L *S. elongatus* PCC 7942 for 72 h in BG-11 containing 100 mM NaHCO_3_ (Gao et al., 2016). Notably, it was demonstrated that the *S. elongatus* + *E. coli* co-cultivation system could provide isoprene titers as high as 0.4 g/L (Liu et al., 2021). The promoter, source of the synthase, and host organism may be reasons for the yield difference. It is also worth optimizing the ratio of the cyanobacteria and *E. coli* for more stable production and operates on higher cellular densities to improve the production per liter of cell culture (Zhao et al., 2011). Designing an effective inoculation approach may lead to considerable enhancement in terms of isoprene titers; however, it also affects the time required to reach satisfactory product levels in S. *elongatus* + *E. coli* co-cultures (Liu et al., 2021).

When the individual strain BL21_*efe_PS^+^* and BL21_*IspS_EG^+^* was cultured in co-culture media with 0.05 g/L sucrose (the sucrose yield of cyanobacteria in 2 days), the production of volatile platform hydrocarbons was lower than that of the consortium. Therefore, there are two possible explanations for these results. First, the gradual sucrose secretion during the co-culturing improved the yield of isoprene, and the consumption of sucrose by *E. coli* may allow the cyanobacteria to secrete more sucrose. Alternatively, cyanobacteria natively produce and secrete other than carbohydrates metabolites that can be metabolized by *E. coli.* Moreover, the higher availability of oxygen thanks to the oxygen evolution reaction can also have a positive impact on the co-culture system when compared with the sucrose supplementation study.

When the individual *E. coli* strain was cultured in consortium media with sufficient sucrose (2 g/L), the production of individual strain BL21_*efe_PS^+^* in co-culture media supplemented with 2 g/L sucrose was 73.93 μmol/L (Co-BG11) and 89.16 μmol/L (M9-BG11), and the production of individual strain BL21_*IspS_EG^+^* in co-culture mediums supplemented with 2 g/L sucrose is 0.38 μmol/L (Co-BG11) and 0.67 μmol/L (M9-BG11). In other words, *E. coli* can produce 37–45 μmol ethylene or 0.19–0.34 μmol isoprene with 1 g of sucrose, which was also used as the energy and carbon source. Cells grew better with sufficient sucrose (the final cell density is OD_600_ = 0.6 with 2 g/L sucrose while cell density is OD_600_ = 0.2 in the consortium system), and the production of ethylene and isoprene per liter culture was higher than that of the consortium system, but the production per cell had little difference. On the one hand, sucrose secreted by cyanobacteria in the co-culture system may be mainly used to produce volatile platform hydrocarbons with a high utilization rate; on the other hand, *E. coli* cells hardly grew with limited sucrose resulting in lower total production per liter culture.

## Discussion

Sucrose yield in this study was 0.14 g/L (E542*_cscB^+^*) and 0.07 g/L (PCC7942*_cscB^+^*; Figure 2). This yield is lower than in previous studies (Hays et al., 2016; Li et al., 2017; Zhang et al., 2020), and it may be influenced by the strain, constructs, and, importantly, the lack of external inorganic carbon supply in the form of highly concentrated CO_2_ or bicarbonate. Consequently, the growth parameters under salt stress in this study are inferior to many of those presented earlier. For example, it took 10 more days to grow from OD_730_ = 0.05 to OD_730_ = 0.8 (Supplementary Figure 2) under a light intensity of ~36 μEm^−2^ s^−1^ in BG11 with 150 mM NaCl and no CO_2_ supplementation than it did in other studies. For instance, PCC7942 cells incubated at 35°C, 2% CO_2_, 150 rpm, ~65 μEm^−2^ s^−1^ light intensity, grew to OD_750_ = 1 in 7 days in BG11 containing 150 mM NaCl (Ducat et al., 2012). Elsewhere, cell density of OD_750_ = 3 was achieved in 3 days in Co-BG11 (106 mM NaCl and other ions), 35°C, 2% CO_2_, 150 rpm, and initial cell density OD_750_ = 0.25 (Hays et al., 2016). All of these indicate that light intensity and CO_2_ supplementation are crucial for faster growth and are significant factors to improve the sucrose secretion efficiency and to provide more sucrose to *E. coli* in the consortium system.

Two kinds of co-culture media were compared for the cell growth, gene expression, and production of volatile hydrocarbon. The results showed that when sucrose was served as the only carbon source, sucrose utilization-related genes (*csc* series genes) showed an obvious upward transcription to provide energy for cell growth, and the volatile platform hydrocarbons could be successfully expressed (Figure 4, Supplementary Figure 3). The transgenic cyanobacterial cells exhibited satisfactory growth and sucrose secretion in both growth media, and its performance in M9-BG11 medium was superior to that in Co-BG11 (Figure 5, Supplementary Figure 4). In the consortium system, both ethylene and isoprene were synthesized, and the maximum yields were 52.71 μmol/l (ethylene production) and 0.75 μmol/l (isoprene production), respectively (Tables 2, 3).

Compared to the productivity in the LB medium, ethylene and isoprene yield per liter of cell culture for individual *E. coli* strain, decreased in the co-culture medium supplemented with 2 g/L sucrose. It may be caused by the inhibition of cell growth and metabolism due to incomplete sucrose utilization. In many studies utilizing sucrose as a shuttle molecule in the consortium, *csc* series genes were artificially regulated (overexpressed *cscA*, *cscB*, and *cscK*, deleted gene for sucrose catabolism operon repressor *cscR* etc.) to improve sucrose utilization in *E. coli* (Weiss et al., 2017; Zhang et al., 2020). It may be worth exploring similar strategies in the future to improve the performance of consortia in the production of volatile hydrocarbons. Under the conditions tested in this study, the consortium producing isoprene showed better yields than that for ethylene production. The result was consistent with the results of RT-qPCR describing gene transcription under sucrose utilization (*IspS* gene mRNA level increased, while *efe* gene mRNA level decreased) caused by different synthesis pathways for the two platform hydrocarbons in *E. coli* (Figure 1). Arginine and α-ketoglutaric acid are the precursors of ethylene synthesis. In LB medium, arginine can be obtained directly from tryptone present in the medium. Meanwhile, sufficient protein and other energy sources can produce enough acetyl-CoA to drive the synthesis of α-ketoglutaric acid. However, in the co-culture medium, sucrose was used as the sole carbon source, which made acetyl-CoA synthesis dependent on glycolysis and simultaneously exploited for amino acid synthesis and other important metabolic pathways, resulting in a decrease of flux to α-ketoglutaric acid. Lower precursor availability resulted in a significant decrease in ethylene synthesis. In contrast, isoprene precursors in *E. coli* BL21 are synthesized *via* the MEP pathway from pyruvate and glyceraldehyde-3-phosphate, mainly derived from glycolysis. Compared with the LB medium, the co-culture system directly provided sucrose to *E. coli*., which was beneficial to the synthesis of isoprene, but not ethylene.

## Conclusion

In this study, artificial consortia comprising *E. coli* and cyanobacteria, using sucrose as carbon and electron shuttle, for the production of volatile hydrocarbons were successfully constructed. The *cscB* gene was successfully expressed in both cyanobacterial strains. The results showed that the sucrose yield of the strains was 0.14 g/L (E542*_cscB^+^*) and 0.07 g/L (PCC7942*_cscB^+^*) under their respective growth temperatures, similar to other studies that did not utilize carbon dioxide supplementation but lower compared to the studies that applied high light and external CO_2_ supplementation. This suggests that sucrose production by cyanobacteria expressing the sucrose transporter is typically carbon limited and an increased flux toward the secreted carbon can be generated using higher inorganic carbon loads and fine-tuning electron supply from the photosystem for carbon fixation and biosynthesis of sucrose. The growth parameters and gene expression of both engineered members of the artificial community were compared and analyzed, suggesting different metabolic responses of the community members depending on the type of product being synthesized. Various combinations of the consortia cultivation parameters were tested and the maximal yields of hydrocarbon production were achieved using 52.71 μmol/L for ethylene in M9-BG11 with E542 and 0.75 μmol/L for isoprene in M9-BG11 with PCC7942. The productivity of co-culture systems was similar to or superior to pure cultures containing an equivalent carbon source, showing the consortium partners’ positive effect on producing these platform chemicals. However, from the applicative point of view, there is still a significant productivity gap that will allow these systems to replace fossil-derived volatile platform chemicals. The areas that could further improve the productivity of such a system include optimization of carbon and light supply, enhanced cellular concentrations, and in the longer scheme formation of hierarchical structures in which different community members could be arranged to facilitate the transfer of carbon between community members.

## Data availability statement

The original contributions presented in the study are included in the article/Supplementary material, further inquiries can be directed to the corresponding author.

## Author contributions

YC: conceptualization, methodology, validation, formal analysis, investigation, data curation, writing—original draft, writing—review and editing, and visualization. FR: methodology, validation, investigation, data curation, writing—review and editing, and supervision. YJ and YZ: investigation and data curation. SZ: supervision and project administration. TB: resources, validation, formal analysis, writing—review and editing, and visualization. SR: methodology and supervision. MD: conceptualization, methodology, resources, validation, formal analysis, investigation, data curation, writing—original draft, writing—review and editing, visualization, supervision, project administration, and funding acquisition. All authors contributed to the article and approved the submitted version.

## Funding

This research was funded by Shenzhen Fundamental Research Program (GXWD20201231165807007-20200806170221001).

## Conflict of interest

The authors declare that the research was conducted in the absence of any commercial or financial relationships that could be construed as a potential conflict of interest.

## Publisher’s note

All claims expressed in this article are solely those of the authors and do not necessarily represent those of their affiliated organizations, or those of the publisher, the editors and the reviewers. Any product that may be evaluated in this article, or claim that may be made by its manufacturer, is not guaranteed or endorsed by the publisher.
