# Supplementary material for: Construction of an artificial consortium of Escherichia coli and cyanobacteria for clean indirect production of volatile platform hydrocarbons from CO2
Source: Front Microbiol. 2022 Oct 21;13:965968. doi: 10.3389/fmicb.2022.965968 (PMC9635338; doi:10.3389/fmicb.2022.965968)
Supplement: Supplementary file 1 [file Data_Sheet_1.DOCX]

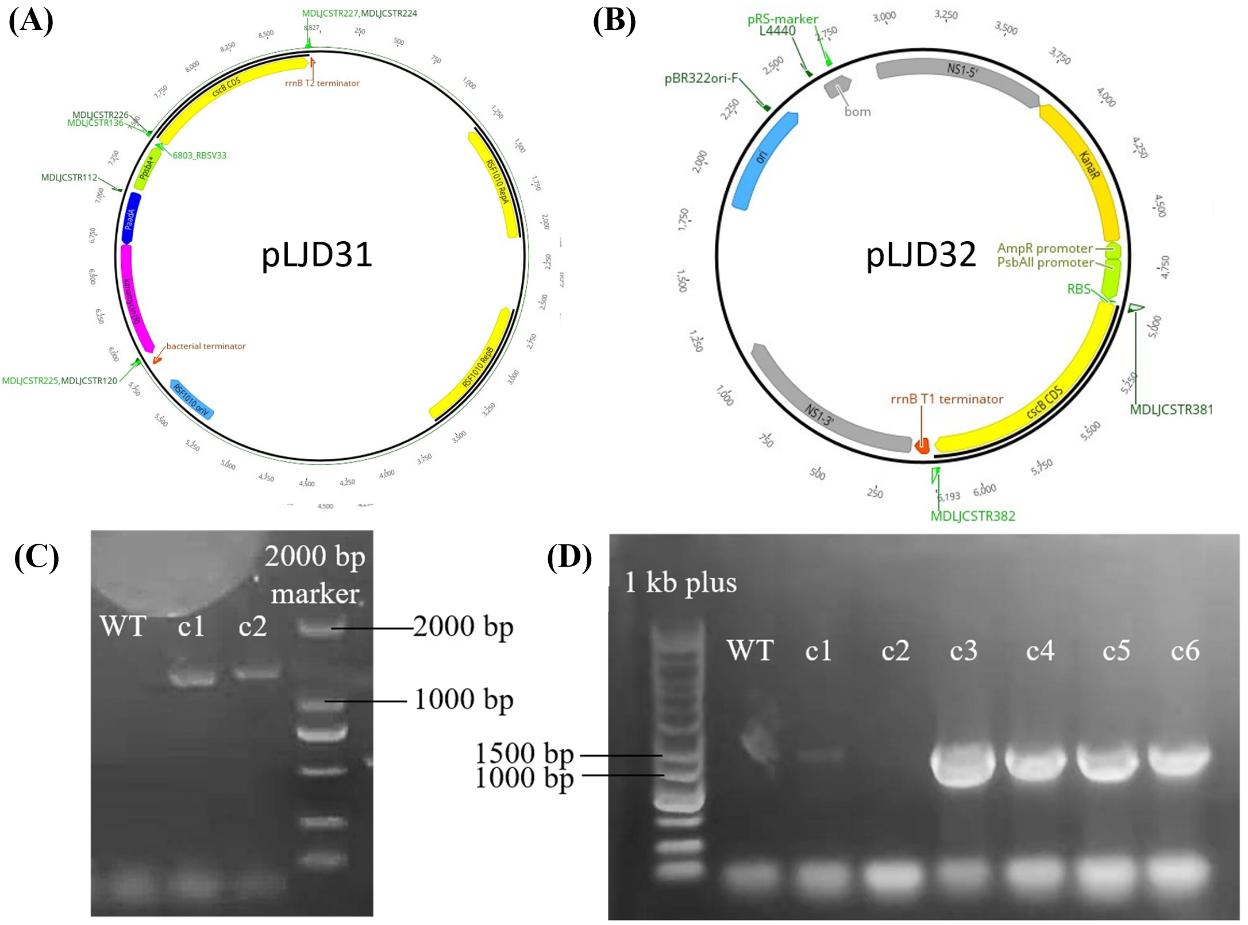
Supplementary Material:

**Supplementary Figure 1.** Map of the plasmids and primers for pLJD31 (for E542) **(A)** and pLJD32 **(B)**. Identification of *cscB* gene in E542_*cscB^+^* (with primer MDLJCSTR226/227) **(C)** and 7942_*cscB^+^*(with primer MDLJCSTR381/382) **(D)**: WT: wild type cyanobacteria strains; c: selected colonies of engineered strains.


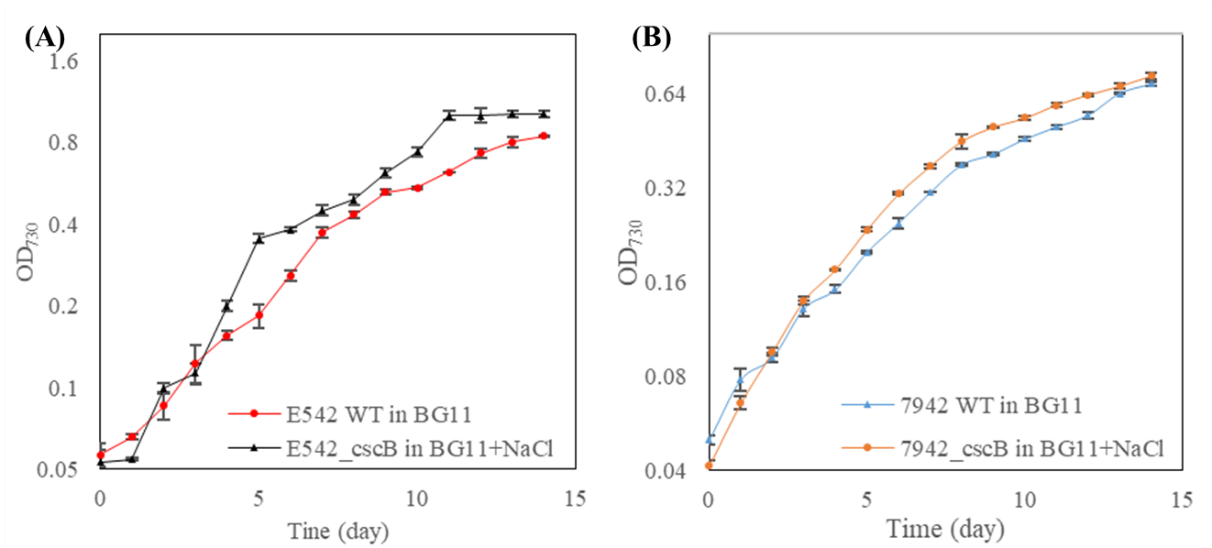


**Supplementary Figure 2.** Comparison of growth curves of the engineered strains of E542*_cscB^+^* (left) and PCC7942_*cscB^+^* (right) in BG11 with 150 mM NaCl with their corresponding wild types (WT) in BG11.


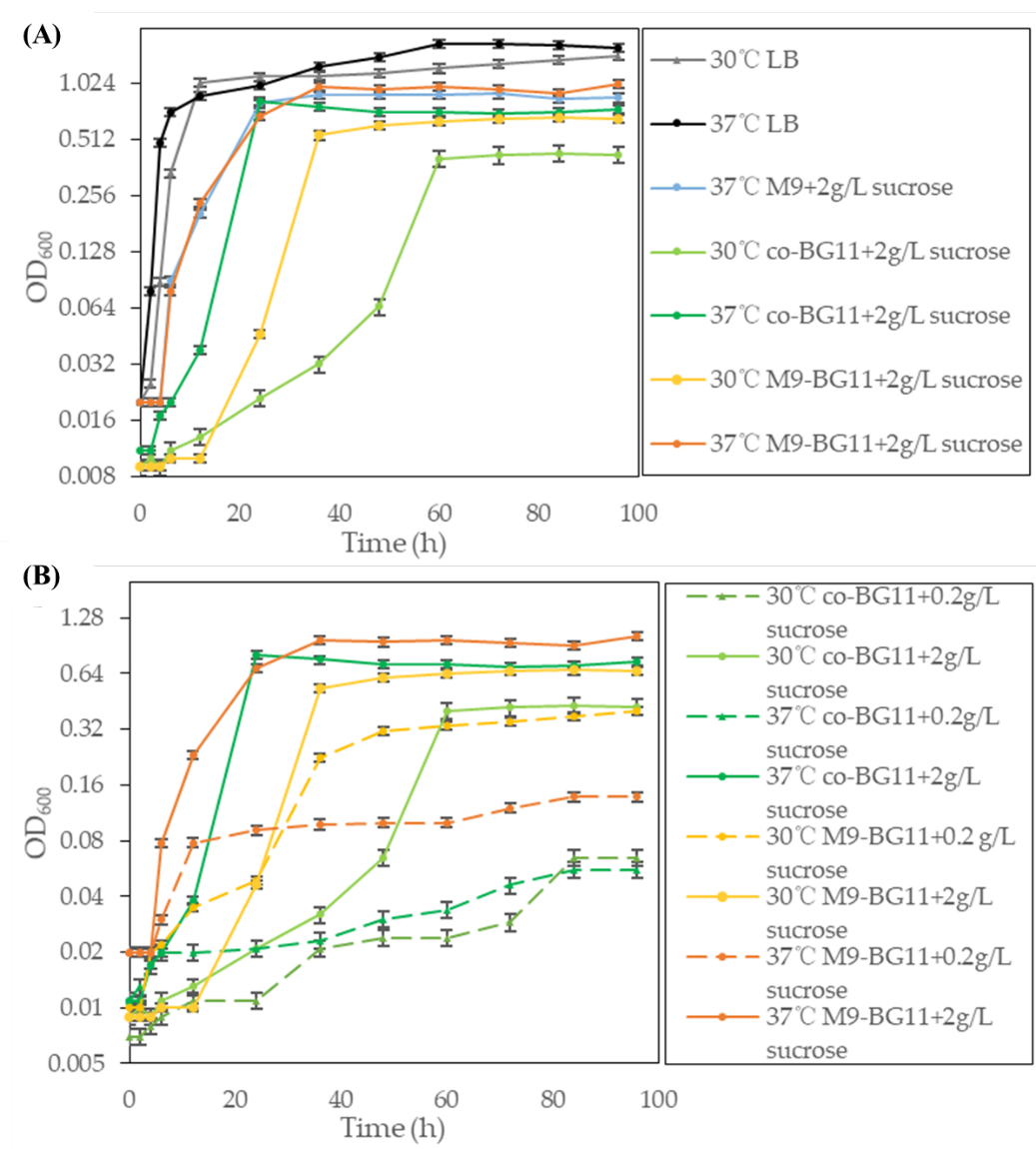


**Supplementary Figure 3.** Growth curves of the BL21_*efe_PS^+^* cultivated across different temperatures in standard E. coli and co-culture media supplemented with 2 g/L sucrose **(A)** and different sucrose concentrations (2 g/L and 0.2 g/L) in co-culture media **(B)**


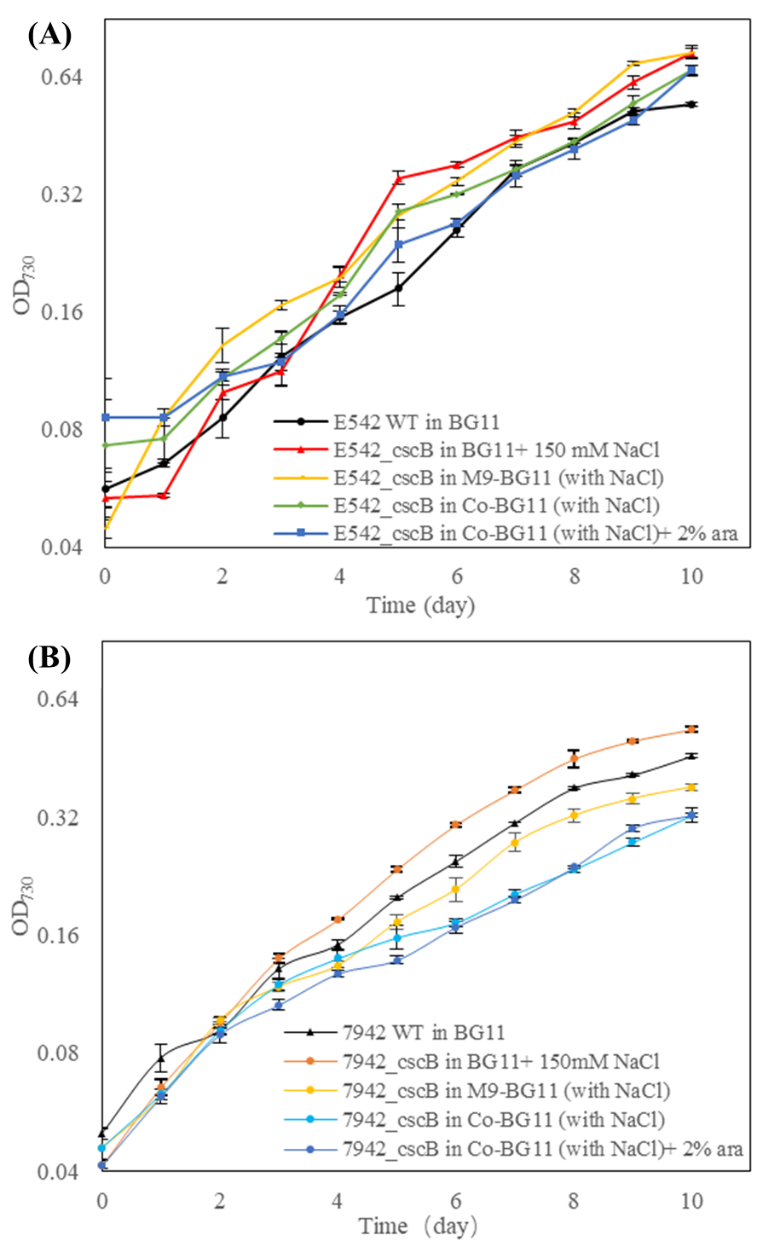


**Supplementary Figure 4.** Growth curves of the engineered strains E542*_cscB^+^* **(A)** and PCC7942*_cscB^+^* **(B)** in co-culture media (with 150 mM NaCl).
